# Individual Monitoring and Occupational Dose Record Management in China: History, Current Status and Perspectives

**DOI:** 10.3390/ijerph13060558

**Published:** 2016-06-03

**Authors:** Hong-Bo Wang, Hai-Tao Yu, Quan-Fu Sun

**Affiliations:** 1 Key Laboratory of Radiological Protection and Nuclear Emergency, National Institute for Radiological Protection, Chinese Center for Disease Control and Prevention, Beijing 100088, China; hongpo212@163.com; 2 Qingdao Municipal Center for Disease Control and Prevention, Qingdao 266033, China; rockfish_1976@126.com

**Keywords:** individual monitoring, occupational dose record management, China

## Abstract

This review paper presents an overview of individual monitoring, as well as the national dose register and dose record management of radiation workers in China. Progress has recently been made on the individual monitoring of radiation workers. A critical analysis of current status and problems in individual monitoring is also presented and necessary future research on individual monitoring, such as the monitoring technology in the form of the ring dosimeters and eye lens dosimeters, is suggested.

## 1. Introduction

The individual monitoring system was established in the nuclear industry sector in the 1950s in China. In 1985, the Regulation on Individual Monitoring of Radiation Workers, together with the Methods of Individual Monitoring for Radiation Workers, was issued by the Ministry of Health. A nationwide monitoring of individual doses has been gradually implemented since 1 December 1985. Individual monitoring is an important part of the radiation health work. It can objectively reflect the level of the dose received by the radiation workers and provide the necessary basis for the radiological protection evaluation and the necessity to follow up on possible acute radiation tissue injuries. Individual monitoring is of great significance to the discovery and improvement of the relevant issues in the field of radiation protection, and to the guarantee of occupational health and safety of radiation workers. The history, current status and perspectives of individual monitoring of radiation workers in China are introduced briefly in this article.

## 2. History

The application of nuclear energy and nuclear technology began in the 1950s in China [1]. With the continuous development of science and technology, radioisotope and X-ray installations have been widely used with important contributions made to the national defense, national economy and medicine and health care. However, while enjoying the benefits brought about by the application of radiation, we must be alert to the risks from radiation exposure, especially damage to man and other living organisms, and pollution to the environment. In order to ensure the health and safety of radiation workers and the general public, the relevant national standard and regulations [2,3] stipulate that individual monitoring should cover all workers involving radiation.

Individual monitoring is not only a kind of routine technical service but also a systematic engineering and technology. Closely concerned with the health of radiation workers, it is one of the most important contents of the health surveillance of radiation workers [1], and an important means of radiological protection. It also helps to evaluate the radiological protection of the radiation workers and improve the level of protection.

Individual monitoring became a relative comprehensive system in the early 1970s. Subsequently, the Nuclear Industry Individual Monitoring and Service Center (NIMSC) was set up in 1986. Individual monitoring for radiation workers was mostly conducted inside the nuclear industry system at that time. The first national meeting on occupational individual dose data statistical analysis and assessment was held in February 1981, summarizing the data of external exposure individual monitoring from 1959 to 1979, encompassing nuclear reactors, reprocessing plant and research institutes, with both preliminary assessment and comparison with data in other countries being carried out.

The policy of reform and openness to the outside was implemented in China in 1978. With the rapid development of the economy, the applications of nuclear and radiation technology have been increasingly used in sectors outside the nuclear industry such as medicine and non-nuclear industries. In order to meet the needs of the radiation protection of workers under the new situation, the individual monitoring and health examination were launched, on a step by step basis, for the radiation workers involved in medicine and industry sectors. 

In 1985, the Regulations on Individual Monitoring of Radiation Workers, together with the Methods of Individual Monitoring for Radiation Workers, were issued by the Ministry of Health. In 1988, the Ministry of Health issued regulations on health management of radiation workers. In 1997, the Ministry of Health re-issued regulations on health management of radiation workers in the form of a Decree of the Ministry of Health (No. 52). In 2007, the Ministry of Health issued Management Methods for Occupational Health of Radiation Workers [3] in the form of a Decree of the Ministry of Health (No. 55), which is still in use at present.

A nationwide individual monitoring has been gradually implemented since 1 December 1985. The technical management of individual monitoring was initially undertaken by Tianjin Institute of Radiation Medicine, Chinese Academy of Medical Sciences prior to 1994, and, afterwards in 2003, by the Industrial Hygiene Laboratory of the Ministry of Health, renamed the National Institute for Radiological Protection (NIRP), Chinese Center for Disease Control and Prevention. 

The Decree of the Ministry of Health of the People’s Republic of China (No. 55) [3] stipulates that NIRP is responsible for assisting the Ministry of Health to formulate procedures and standards of qualification examination and approval of individual monitoring services, organizing the implementation of the national individual monitoring quality control and technical training, and summarizing and analyzing the national individual monitoring data.

The Law of the People’s Republic of China on Prevention and Control of Occupational Disease [4], which was adopted in 2001 and in effect since 1 May 2002, and amended in 2011, stipulates that employers must ensure that all the workers involving radiation exposure wear personal dosimeters. 

The State Commission Office for Public Sector Reform (SCOPSR) re-assigned the Ministry of Health to take the responsibility to approve the individual monitoring service providers in October 2010.

## 3. Current Status

### 3.1. Radiation Units and Radiation Workers and Their Distribution

There are almost 70,000 radiation employers with about 350,000 radiation workers in China at present. About 60%–70% of the total is involved in medical diagnosis (X-ray and nuclear medicine imaging) and radiation therapy. In addition, 10% are in the sectors of industrial radiography and irradiation installations, 10% are in education and research sectors, and the remaining 10% are in nuclear industry sectors. Among these workers, 223,000 radiation workers are working in 53,000 medical diagnoses and radiation therapy institutions.

In the non-nuclear industrial sectors, radiation workers are distributed mainly in eastern and central China. In the nuclear industrial sector, most of the radiation workers are working in uranium mines. There are more than 10,000 workers in nuclear power plants, which are expected to greatly increase in the future. At present, in addition to the operating China Experimental Fast Reactor (CEFR) at the China Institute of Atomic Energy (CIAE), there are 27 nuclear units in operation, 23 under construction, and some others pending construction licenses or in the early preparatory phase.

After years of efforts, the coverage of individual monitoring has risen steadily, currently at about 90% [5]. The difference of the coverage from region to region and industry to industry is obvious. Depending on the differences of emphasis on individual monitoring of the radiation units and the differences of law enforcement dynamics of the health supervision departments in different regions, the coverage of individual monitoring of radiation workers in eastern China is higher compared with that in western China on the whole. Being the first sector to conduct individual monitoring in China, the nuclear industrial sector has always attached great importance to individual monitoring, and individual monitoring of radiation workers in the nuclear power plant is fully covered. Average annual effective dose of radiation workers from 1985 to 2012 has shown a significant downward trend. The coverage of individual monitoring in Shanghai and Beijing is more than 95%. 

### 3.2. Laws and Regulations Governing Individual Monitoring

The laws and regulations governing individual monitoring are listed in Table 1.

Under Qualification Conditions of Individual Monitoring Services for Radiation Workers [7], the service providers should meet the following requirements:

(1) Having legal representative qualifications or legal-authorized qualification;
(2) Being capable of carrying out corresponding technical service work independently;
(3) Having a permanent office and workplace and working conditions;
(4) Having a reasonable post setting and well-defined responsibility scope;
(5) Having robust quality assurance system.

Personnel should meet the following requirements:

(1) Being fit for management, technique and quality control required by the programs;
(2) Being familiar with the relevant laws, standards and documents as well as quality management manuals;
(3) Technical director of individual monitoring with junior college diploma or above, or the intermediate technical titles or above and having experiences in related work more than three years;
(4) Being qualified through training and examination;
(5) Total number of operators is not less than three.

Instruments and equipment should meet the following requirements:

(1) Having corresponding instruments and equipment required for carrying out technical service of individual monitoring;
(2) Type, quantity, performance, range and precision of the instruments and equipment are capable of meeting the needs of the work, and the instruments and equipment can run well;
(3) Being regularly subject to metrological verification;
(4) Having a complete set of operating procedures.

Under Management Methods for Occupational Health of Radiation Workers (MMOHRW) [3], the provincial health administrative departments are responsible for the qualification examination and approval of individual monitoring services. When applying for qualification of radiological health technical services, the following materials shall be submitted to the health administrative departments: application form; the legal representative qualification certificates materials; the brief introduction to the applicant; the directory of quality management handbook and procedures document; the list of professionals and technical workers; the certificates of technical titles and certificates of training and examination; lists of related equipment; certificate of workplace use; and certificate of meteorology authentication. 

The qualification certificate of radiological health services is valid for four years. Change and extension of qualification certificate are allowed if necessary. Radiological health service units can provide the trans boundary technical services across the administrative areas but shall submit the application, for the record, to the local provincial health administrative department and accept the possible supervision and inspection. 

GBZ 207-2008 [8] specifies the performance testing, evaluation and requirements of routine performance testing, as well as the quality control of performance testing of external exposure individual dose system.

### 3.3. Individual Monitoring Services Providers

There were 190 individual monitoring services in 2009, and in the year of 2012, the number increased to 203. CDCs (Centers for disease control and prevention) and institutions of prevention and treatment of occupational disease represent the overwhelming majority of the monitoring services. Other monitoring services are distributed in the nuclear industry, commercial companies, universities, environmental protection agencies, *etc.* The distribution of the individual monitoring services is not uniform throughout the country. Most of the monitoring services are distributed in eastern China. Jiangsu and Shandong possess the most individual monitoring services among 34 provincial administrative regions. 

At present, the vast majority of monitoring services can only provide the monitoring data of annual effective dose from external gamma and X-ray exposure [9], and are unable to provide adequate monitoring of alpha- and beta-radiation, and dose to lens/hands and internal exposure. Three services can monitor more than 10,000 workers annually, 10 services can monitor 4000 to 10,000 workers annually, 30 services can monitor 1000 to 4000 workers annually, and the others can monitor less than 1000 workers annually. Internal individual monitoring has not been implemented in most areas of China. This is especially true for medical radiation workers.

Generally, there are a small number of individual monitoring services in developed countries, which are characterized by their big size and large amount of tasks [10]. However, most Chinese units feature a small size and small amount of work. Furthermore, most units are unable to undertake comprehensive tasks of individual monitoring due to weak capacity [11]. 

### 3.4. Individual Monitoring Personnel, Equipment and Period

The number of staff in each individual monitoring service varies from 1 to 16, with an average of 3.8 in each service. A national survey [12] demonstrated that radiological health professionals with middle professional titles and primary professional titles account for 42.6% and 38.2% of the total, respectively. Professional title series in China include primary professional titles, middle professional titles and high professional titles. Education backgrounds with junior college and technical secondary school account for 36.6% and 34.5% of the total, respectively. Radiological health professionals majored in preventive medicine account for 45.7% of the total, which is the largest proportion.

An investigation [11] conducted in 2007 indicated that 87.5% of thermoluminescence detectors (TLD) readers from 56 individual monitoring services were domestic equipment and that 92.44% of the individual monitoring services were using Lithium Fluoride (Mg, Cu, P) thermoluminescence detectors in the form of powder or sheets. Irradiation equipment can be shared between services for screening of Lithium Fluoride (Mg, Cu, P) thermoluminescence detectors. In 2012, 7% of the services that participated in the national intercomparison program failed to pass the test.

Dosimeters are calibrated to measure Hp (10). Workers wear under-apron dosimeters on the chest when working in order to obtain the external dose, and the criterion used for external dose monitoring is GBZ 128-2002 [2].

The individual monitoring generally takes a longer time period of about three months in most areas of China. Regulation [3] requires that the individual monitoring period should be one month in general, and should not exceed three months at a maximum. Although fading of dosimeter signal due to long-time wearing would seldom affect the monitoring results, it is unable to facilitate the timely discovery of any abnormal exposure situations for high dose workplaces.

### 3.5. Dose Record Management and National Dose Register

There was no statistical and reporting system available before the 1980s. With the policy of reform and openness in the 1980s, nuclear and radiation technology applications were more frequently used in medicine, industry and other sectors. In 1979, the Ministry of Health and other ministries issued a revised MMOHRW, reaffirming the establishment of license registration and radiation event reporting systems. In 1985, an individual monitoring system was formally established. With the popularity of computer and information technology, the health and other relevant departments made a useful exploration for the gradual establishment of health monitoring statistics automation, including radiological health. 

The Service Center of Occupational Exposure Dose Management for Nuclear Industry was set up in 1985, under the auspices of the China National Nuclear Corporation (CNNC), with the responsibility for development of data management systems, collection and analysis of monitoring data from nuclear power plants, and submission of annual assessment reports. In 1998, the Industrial Hygiene Laboratory of the Ministry of Health developed software for data processing and file management of individual monitoring for radiation workers (DPFM), in an attempt to unify the data management of individual monitoring in the medical field. Problems existing in the software of individual monitoring information management system were discussed in selected references [13,14,15]. 

Health supervision was separated from technical service due to the reform of the system of health and epidemic prevention. After effective control of SARS (Severe Acute Respiratory Syndrome) in 2003, more attention was paid to collecting information for each case associated with disease control and prevention as well as to direct reporting to higher authorities by use of computer information technology. In October 2003, the Chinese Center for Disease Control and Prevention began to run an information system of the health hazard monitoring, with a direct-reporting-card of individual monitoring being included.

Relevant institutions have made some attempts in preparation for radiation health reports, especially individual monitoring data reports. However, the effort was not very successful for various reasons. Several factors led to difficulty in system extension, such as a lack of professional quality control measures, unreasonable data reporting and rough demand analysis. A large number of papery records were hardly regarded as a scientific basis for radiation health decision-making. Reporting format of data did not meet the requirements of International Atomic Energy Agency (IAEA) and United Nations Scientific Committee on the Effects of Atomic Radiation (UNSCEAR).

As pointed out by the IAEA Occupational Radiation Protection Appraisal Service (ORPAS), when visiting China in August 2004, a data reporting system of individual monitoring of radiation workers in China should be completely reconstructed. It was strongly suggested that China establish a national central database and improve quality control and quality assurance of individual monitoring. Since 2005, funded by IAEA, China began to establish the occupational health management system of radiation workers inside the system of Ministry of Health under the framework of the IAEA CPR (CPR/9/037). As a result, the China Register of Radiation Workers (CRRW) was officially released by the Ministry of Health on 25 November 2009. The system has authorized 212 users, including 180 individual monitoring services and 32 supervision departments. By 2014, there had been 2 million monitoring records in the central database of 330,000 workers belonging to 35,000 radiation users countrywide. 

The database information of the system of CRRW includes individual monitoring services, equipment, radiation users (employers), radiation workers (employees), doses, occupational health management, *etc.*

**Figure 1:**
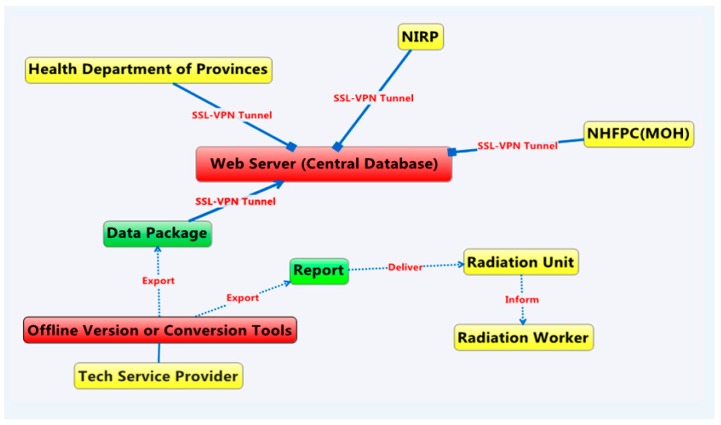
Structure of China Register of Radiation Workers (CRRW) System: PC version + web version.

From Figure 1, we can see that CRRW consists of two parts, an offline version and online version, respectively. The offline version is comprised of seven function modules (new, dose data, reports, query, system management, export and exit). Uniformed annual reports, periodic reports, and suspicious data inspection sheets can be generated by using the offline version. The online version is comprised of six function modules, such as query, statistical analysis, data management, report cards, system management and exit. Summary tabulations can be generated by using the online version [16].

Name, age, sex, working unit, unique identification number, occupational exposure category, quarterly and annual effective doses among others are uploaded by each monitoring service and kept in the register. Backup systems including optical disks and hard disks in different places are used to store the dose records safely.

The Ministry of Health issued a notification in 2009 to enable the individual dose management system and request qualified agencies to upload the relevant information timely. In addition, the Ministry of Health commissioned the NIRP to be responsible for technical service, maintenance, management of the individual dose management system, the local technical guidance, *etc.*

According to the notification of the Ministry of Health, individual monitoring services began to upload relevant information from the end of 2009. The quality problem of the uploaded information includes: the lack of professional category and improper filling out of the professional category; the small-scale institutions changing the name of the entity frequently, multiple institutions being identified by the system; mistakenly filling out beginning and ending dates; and uploading the test results as the final dose report owing to unfinished large dose verification.

Due to the lack of clear guidance, the process of large dose verification varies throughout the country, and the phenomenon of long verification cycles and low return rates is very common. As a result, many unfinished large dose test results are uploaded as the final dose, with dosage value overvalued. 

### 3.6. Trend of Individual Monitoring Coverage and Annual per Capita Effective Dose 

As can be seen from Table 2, the number of workers occupationally exposed to radiation in medical applications and monitoring coverage is rising on the whole. Annual per capita effective doses are declining, from 2.15 mSv (1986–1990) to 0.53 mSv (2009–2013). From Table 3, we can see that the individual monitoring coverage in industrial applications did not increase obviously during 1997 to 2000; however, annual per capita effective doses in each occupation category are declining. It probably benefits from the significant improvement of occupational protection level in China’s medical and industrial sectors. In addition, the decrease in annual per capita effective dose may also probably be an effect of the extension of the group to persons who are less exposed than those monitored earlier.

The number of workers who should be monitored in Table 2 and Table 3 were obtained from the relevant statistical report forms on radiological health formulated by the Ministry of Health. The number of people who should be monitored were identified according to the relevant national standard and regulations [2,3]. That is to say, the number of workers who should be monitored is only the number of radiation workers.

## 4. Perspectives

### 4.1. Individual Monitoring in Nuclear Medicine and Interventional Radiology to Be Strengthened

Attention should be paid to the health of nuclear medicine and interventional radiology workers in hospitals. By the end of 2013, a total of 8678 workers were working in the field of nuclear medicine in China [17]. There were more than 50,000 medical workers who were engaged in the clinical work of interventional radiology by 2006 [18], and newer and more accurate data have not been seen. According to a cross-sectional survey [19], the risk of subcapsular opacity of the eye lens among interventional radiology and nuclear medicine workers increased significantly compared with conventional radiologists in the same hospitals. Several studies [18,20] have also reported that such symptoms as detection frequency of neurasthenia, skin changes, abnormal white blood cell count, unstable chromosome aberration frequency and micronucleus frequency increased in the interventional radiology group, as compared with other groups.

Efforts should be made to strengthen research on the monitoring technology of the ring dosimeters and eye lens dosimeters, carrying out monitoring and evaluation of extremity doses and eye lens doses of interventional radiology and nuclear medicine workers [5], monitoring the doses and health of workers in interventional radiology and nuclear medicine more closely, and carrying out monitoring of internal exposure and estimation of doses for nuclear medicine staff.

### 4.2. Regulations and Standards to Be Improved

GBZ 128-2002 [2] stipulates that reference dosimeters that can provide background information should be used when monitoring, but there are no unified and specific requirements in actual monitoring practice. In the actual monitoring work, the natural background radiation data is not processed in a consistent approach [11]. The background radiation should be subtracted from the measured doses when calculating the doses, but the situation is not the case. In China, about 95% of radiation workers are receiving an annual effective dose of less than 5 mSv. There is a lack of formal and unified procedure to handle the “big dose” and assign the “nominal dose”. More efforts are needed to improve our individual monitoring in the future. 

It is expected that the Ministry of Health should formulate clear specification of large dose verification procedures as soon as possible. Unified and specific requirements on how to obtain the background radiation should be also formulated, and besides, it is expected that GB 18871-2002 [21] will be upgraded based on the 2014 Basic Safety Standards [22].

### 4.3. Miners and Aircrews to Be Covered 

NORM (naturally occurring radioactive material) and TENORM (technologically-enhanced naturally occurring radioactive material) in mines have become more and more important in radiological protection in China. At present, there are about ten million miners working in more than 8000 state-owned mines and 100,000 other mines, including coal mines. In total, there are six million coal miners and four million other miners. It is estimated that radon concentration in 15% of the underground mines exceeds 1000 Bq/m^3^ [23]. 

Aircrews have not yet been regarded as radiation workers. There are about 30,000 to 40,000 aircrew members in China. It is estimated that radiation doses to pilots and stewards are 1.53 mSv/a and 1.90 mSv/a, respectively [24]. Stewards of international routes include more young females, some of whom have not had a child. Particular attention should be paid to breast cancer, leukemia and the impact of cosmic rays on the fetus.

Efforts should be made to expand the scope of radiation workers, incorporate the aircrew and high radon exposure group (such as certain underground miners and other workers) into the occupational exposure range so that their health can be protected, and develop personal radon monitors for high radon exposed miners.

### 4.4. Safety Awareness of the Radiation Workers to Be Raised

With the improved ionizing radiation installation and the strengthened radiation protection measures in recent years, the radiation dose to radiation workers is decreasing significantly. However, there exists carelessness in work among some radiation workers. Some radiation workers are unwilling to wear personal dosimeters or simply put them in a drawer. Some radiation workers think that the monitoring results are too low and there is no practical significance, so they want to test the sensitivity of the dosimeters and deliberately place them under the X-ray beams, making them irradiated. 

Radiation protection awareness of staff needs to be further strengthened. Relevant agencies and organizations should make efforts, such as launching promotional activities, to make them aware of the importance of radiation protection.

### 4.5. Supervision and Enforcement to Be Strengthened

Efforts should be made to improve the supervision and enforcement of radiation health, give necessary administrative punishment to those who are involved in intentional exposure, artificial exposure, or refuse to carry out the required individual monitoring so as to correct problems existing in the management of individual doses promptly, and provide legal guarantee for long-term and standardized implementation of individual monitoring.

## 5. Conclusions

The number of radiation workers in China has been increasing in recent years. Accordingly, their radiological protection and health conditions are receiving more and more attention. Individual monitoring work that is conducted normatively and continuously can not only provide basic data for optimization of radiation protection, but also provide an important basis for improving radiation protection measures and doing research on radiation hazards. In addition, the data of individual monitoring can be used for epidemiological studies and legal purposes [25]. Over the past 30 years, China’s individual monitoring work has made significant progress. In the future, China should attach more importance not only to development of new technologies of dose monitoring and supervision of the wearing of personal dosimeters, but also improve the radiation protection awareness of radiation workers.

## Figures and Tables

**Table 1:** Laws and regulations governing individual monitoring.

Laws and Regulations	Articles	Specific Contents
Law of the People’s Republic of China on Prevention and Control of Occupational Disease [4]	Article 26	Radioactive workplace and/or radioisotope storage and transport device shall be equipped, by its operator, with necessary protection equipment and alarm devices, ensuring that workers involving radiation exposure must wear personal dosimeters.
Regulations on Safety and Protection of Radioisotopes and Rays Installations [6]	Article 29	A producer, distributor and/or user of radioisotope and radiation generator shall, in compliance with the relevant national regulations on individual monitoring and health management, perform individual monitoring and occupational health examination of radiation workers for production, distribution and use of radioisotope and radiation generator and establish individual dose files and occupational health surveillance files.
Management Methods for Occupational Health of Radiation Workers [3]	Articles 11 to 17	The detailed requirements of the individual monitoring period, individual monitoring file, wearing of dosimeters, qualification review of individual monitoring services, delivery of the reports, and report procedure of individual monitoring results.
Qualification Conditions of Individual Monitoring Services for Radiation Workers [7]	Articles 1 to 3	Requirements for service providers, personnel, instruments and equipment.

**Table 2:** Dose levels of occupational exposure in medical applications (1986–2013).

Period	Workers Should be Monitored/Thousand	Workers Actually Monitored/Thousand	Individual Monitoring Coverage/%	Collective Effective Dose/(Man·Sv)	Annual per Capita Effective Dose/mSv
1986–1990	107.4	19.8	18.4	231	2.15
1994–1995	89.1	34.7	38.9	131	1.47
1996–2000	114.7	59.7	52.0	164	1.43
2002–2005	150.0	50.3	33.5	171.0	1.14
2009–2013	189.2	159.4	84.2	100.6	0.53

**Table 3:** Dose levels of occupational exposure in industrial applications (1986–2000).

Occupation Category	Period	Workers Should be Monitored	Workers Actually Monitored	Individual Monitoring Coverage/%	Collective Effective Dose/(Man·Sv)	Annual per Capita Effective Dose/mSv
Industrial Detecting	1986–1990		4270		14.1	1.64
	1994–1995		7470		22.4	1.42
	1996–2000		10200		22.2	1.18
Industrial irradiation	1997	1327	752	56.7	0.668	0.89
	1998	1370	796	58.1	0.811	1.02
	1999	1113	654	58.8	0.643	0.98
	2000	1396	827	59.2	0.519	0.63
operation of the accelerator	1997	989	739	74.7	0.473	0.64
	1998	1187	722	60.8	0.413	0.57
	1999	975	553	56.7	0.224	0.41
	2000	790	437	55.3	0.15	0.34
Others	1994	16032	7528	47	9.65	1.28
	1995	15378	7408	48.2	11.6	1.57
	1996	18051	9641	53.4	12.3	1.28
	1997	23638	12108	51.2	14.6	1.2
	1998	23999	12713	53	15.2	1.2
	1999	22862	12227	53.5	16.5	1.35
	2000	19999	12696	63.5	13.8	1.09
